# Non-Crop Host Sampling Yields Insights into Small-Scale Population Dynamics of *Drosophila suzukii* (Matsumura)

**DOI:** 10.3390/insects9010005

**Published:** 2018-01-03

**Authors:** Johanna E. Elsensohn, Gregory M. Loeb

**Affiliations:** 1 Department of Entomology and Plant Pathology, North Carolina State University, Raleigh, NC 27695, USA; 2 Department of Entomology, Cornell University, Ithaca, NY 14456, USA; gme1@cornell.edu

**Keywords:** *Drosophila suzukii*, non-crop host, invasive species, population dynamics

## Abstract

Invasive, polyphagous crop pests subsist on a number of crop and non-crop resources. While knowing the full range of host species is important, a seasonal investigation into the use of non-crop plants adjacent to cropping systems provide key insights into some of the factors determining local population dynamics. This study investigated the infestation of non-crop plants by the invasive *Drosophila suzukii* (Matsumura), a pest of numerous economically important stone and small fruit crops, by sampling fruit-producing non-crop hosts adjacent to commercial plantings weekly from June through November in central New York over a two-year period. We found *D. suzukii* infestation rates (number of flies emerged/kg fruit) peaked mid-August through early September, with *Rubus allegheniensis* Porter and *Lonicera morrowii* Asa Gray showing the highest average infestation in both years. Interannual infestation patterns were similar despite a lower number of adults caught in monitoring traps the second year, suggesting *D. suzukii* host use may be density independent.

## 1. Introduction

Many agricultural pests are highly polyphagous and exploit multiple crop and non-crop host plants throughout their life cycle [1,2,3,4]. Temporally unstable food and reproductive host resources necessitate insect movement within and between crop and non-crop areas [2]. As such, polyphagous insects take advantage of the specific phenologies of their hosts to increase local population densities, which can lead to multiple and overlapping generations. *Drosophila suzukii*, the spotted wing drosophila, is a prime example of such a polyphagous, mobile pest. *D. suzukii* is an invasive pest of several major stone (e.g., cherry, peach, plum, and apricot) and other soft-skinned fruit (e.g., caneberry, grape, strawberry, and blueberry) crops worldwide [5,6,7,8]. Without adequate control measures, *D. suzukii*-related damage can have significant economic impacts [8,9,10]. This fly has a well-established host range of non-crop plants [11,12] that can serve as population sources for future infestation events. Diurnal movement between crops and adjacent natural habitats [13,14,15] indicate *D. suzukii* populations may inhabit and exploit multiple host types simultaneously, possibly affecting the effectiveness of on-farm pest management strategies.

The timing and extent to which *D. suzukii* utilize non-crop resources is not well understood and likely varies on a regional basis. This is an especially pertinent question for the northern range of *D. suzukii*, as cold winters may kill off a subset of overwintering adults [16,17] and scant information exists on what happens after *D. suzukii* emerge from overwintering sites before adequate fruit hosts ripen, but see [18,19]. *D. suzukii* can potentially exploit locally-available springtime fruiting non-crop hosts in northern temperate regions to increase adult population levels that later infest summer-fruiting cultivated hosts. If these flies begin their annual infestation cycles in non-crop plants first, those plant species could act as sentinels for early season monitoring and predict infestation risk for growers. At the end of the growing season in northern latitudes, most commercial fruit production ends in October. However, *D. suzukii* may continue to infest available non-host crops before going into reproductive diapause, which is brought on by a number of factors, including cooler temperatures and shortened day length [20,21,22].

Many fruit crops are perennial, long-lived plants. Once planted, these trees and bushes remain stationary while the surrounding ecosystem is dynamic. When a polyphagous pest such as *D. suzukii* arrives in a perennial agroecosystem, growers need responsive management strategies to protect their crops. Understanding how non-crop areas adjacent to commercial fruit crops can affect infestation risk throughout the fruiting season can help to inform management. This current study complements other studies that assessed non-crop host use at snapshot or periodic intervals (e.g., [11,12,23]). Focusing on a fine-scale, season-long, multiyear, non-crop host sampling in various cropping systems may provide a better sense of how *D. suzukii* use alternative non-crop hosts as a function of time of year and host availability.

## 2. Materials and Methods

The survey was conducted from June to November in 2013 and 2014. Candidate non-crop hosts were sampled weekly at eight commercial farms in the Finger Lakes region of New York, U.S.A., excepting one week in 2013. Seven farms grew *D. suzukii*-susceptible crops (e.g., raspberries, cherries), while one location—a cornfield—was used as a comparison site. Wild-growing plants producing soft-skinned fruit adjacent to crop hosts were chosen for inclusion in this study. Ideally, chosen plant species were abundant enough to allow for replication within and across sites while individual plants produced a fruit set large enough to allow for multiple sample collections. Up to five samples (each from a distinct individual) of sound, ripe fruit (i.e., no apparent damage) per plant species were collected from each sampling location weekly depending on availability. For non-*Rubus* spp., we collected up to 100 individual fruits per sample. For *Rubus*, we collected 50 fruits/sample, due to their larger size and weight. While *D. suzukii* can oviposit into under-ripe fruit [24,25], we chose to focus only on the ripe stage as those fruit are at the end of their ontogenesis and can be removed from the plant without sacrificing development. Furthermore, the highest proportion of adult flies emerging from differently aged fruit is from the ripe stage [25]. As such, sampling began when ripe fruit was first observed and continued until senescence or fruit became overripe. The number of samples collected at each site declined toward the end of each species’ fruiting season as the number of individual plants with available fruit decreased.

Fruit samples were placed into individual plastic bags and brought back to the lab for processing. Fruit number and weight were recorded before being transferred to a 473.2 mL plastic container (Fabri-Kal Corp., Kalamazoo, MI, USA). Containers were modified with no-see-um mesh (Outdoor Wilderness Fabrics, Cladwell, ID, USA), venting the bottom and top to allow airflow and prevent moisture buildup. The ventilated containers were set into a 946.4 mL (Fabri-Kal Corp., Kalamazoo, MI, USA) container of the same circumference as the sample cup to collect any liquid released from the fruit. Samples were held at room temperature (21 °C) for two weeks, and monitored for fly development three times per week, for a total of six collection points. All visible pupae and flies were removed at each collection point. Pupae were placed in petri dishes lined with a moistened paper towel square and allowed sufficient time to eclose. Using a dissecting microscope, collected flies were identified as *D. suzukii* male or female, or other *Drosophila* species.

The following plants, identified using regional reference materials [26], were sampled for the entirety of their fruiting season: silky dogwood, *Cornus amomum* Miller (Cornaceae); Morrow’s honeysuckle, *Lonicera morrowii* Asa Gray (Caprifoliaceae), and its hybrids; American pokeweed, *Phytolacca americana* L. (Phytolaccaceae); European buckthorn, *Rhamnus cathartica* L. (Rhamnaceae); blackberry, *Rubus allegheniensis* Porter (Rosaceae), and its hybrids; black raspberry, *Rubus occidentalis* L. (Rosaceae); and bittersweet nightshade, *Solanum dulcamara* L. (Solanaceae) (Table 1). In 2013, but not 2014, the following species were sampled: gray dogwood, *Cornus racemosa* Lamarck (Cornaceae); black cherry, *Prunus serotina* Ehrhart (Rosaceae); choke cherry, *Prunus virginiana* L. (Rosaceae); red raspberry, *Rubus idaeus* L. (Rosaceae); and European cranberrybush, *Viburnum opulus* L. (Adoxaceae). These plants were not sampled in 2014 because either they were found in low abundance or were not infested in 2013 (Tables S1 and S2).

At each site, *D. suzukii* presence was monitored using two clear deli cup traps with 12.5 mm diameter entry holes placed approximately 25 mm from the top of each cup. A yeast, sugar, apple cider vinegar, whole-wheat flour, water mixture was placed in a small insert (118 mL specimen cup, Coviden, Mansfield, MA, USA) within the container, covered with mesh, and surrounded by an apple cider vinegar, ethanol and surfactant drowning solution [27]. The two traps were hung ~1 m off the ground in the canopy of plants not known to be associated with *D. suzukii*, approximately 1 m from a known non-crop host, and at least 10 m from each other. In 2013, traps were placed at all sites, while in 2014, trapping was done at six of the eight sites. Trap catch was collected and bait redeployed each week. Contents of the traps were drained from the drowning solution and examined for *D. suzukii* and other *Drosophila* spp. using a dissecting scope. A subsampling procedure was implemented for any trap catch weighing more than 2 g after draining, whereby 10% by weight of the trap catch was randomly removed, counted in its entirety, and used to estimate total *D. suzukii* numbers. For catches greater than 15 g, a 5% subsample was counted. This methodology was verified by comparing subsample estimates to the full count of the entire sample for five trap collections. The subsample procedure has ~5% margin of error.

For analysis, the total collection period was summarized by week and month and averaged across all sites. The comparison cornfield site was included with all other sites, as no major differences in infestation were observed. To control for differences in fruit size and weight among the different host fruits, a standardized calculation was made using the mean number of flies reared per kilogram of fruit collected. Pupae that did not produce adults were excluded from the data set. An analysis of variance (ANOVA) was conducted using R software (v3.2.2) to look for the effect of month and year on flies per kilogram (hereafter, infestation rate) [28]. Infestation rate values were restricted to samples from which adults emerged and log + 0.0001 transformed to satisfy assumptions of normality. For trapping, the number of *D. suzukii* caught per week was averaged across all sites. An ANOVA to compare trap catches between years was conducted on log + 0.001 transformed *D. suzukii* counts. 

## 3. Results

### 3.1. Seasonal Host Use

**Figure 1:**
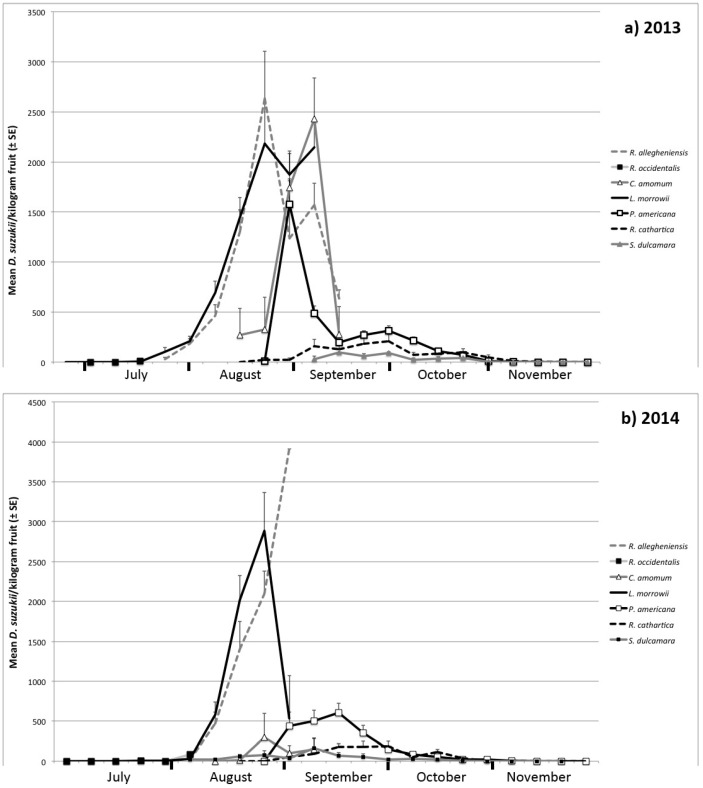
(**a**) Weekly infestation rates of non-crop host plants in 2013; and (**b**) weekly infestation rates of non-crop host plants in 2014. Sampling stopped at the end of the year after two consecutive weeks of no infestation.

In general, the pattern of infestation between years was similar—no to low infestation in June and July, a steady increase and peak in August, followed by a sharp decline beginning in mid-September through the end of November (Figure 1). *Lonicera morrowii* and *Rubus allegheniensis* were the plants most heavily infested within and between years (Table 2). Plants sampled during the first three weeks of spring fruit availability were found to be uninfested, and the first infestations of the year were found in *L. morrowii* and *R. occidentalis* simultaneously. The percent of samples in which at least one *D. suzukii* adult emerged generally peaked when infestation rate for that species was high. Weekly sampling showed a large peak of emerging adults collected from fruit sampled in late August, but a smaller peak that occurred in September in those plants with a later fruiting phenology. The overwhelming majority of flies emerging from all fruit were *D. suzukii*, with 2.7% being other Drosophilidae (417/15,287 total flies for both years), and a male:female *D. suzukii* ratio of 0.88:1.

**Figure 2:**
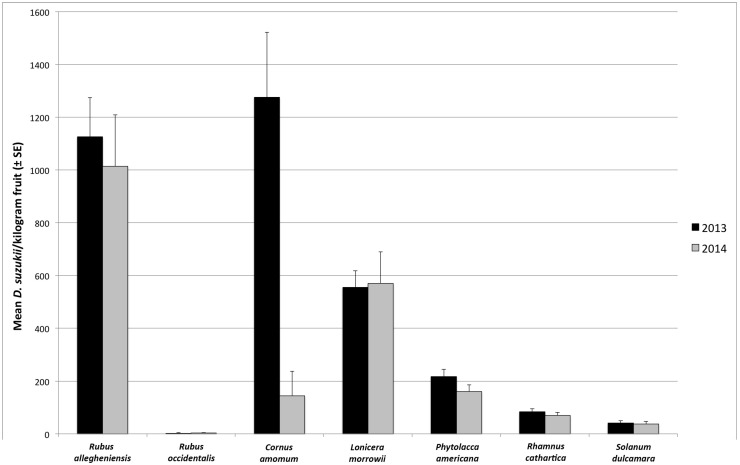
Annual mean number of *D. suzukii* adults (±SE) emerging from collected samples.

There was a higher level of variation in infestation rates between months (*p* < 0.001, *F*_4, 358_ = 38.69) than between years (*p* = 0.226, *F*_1, 361_ = 1.47), with no significant interannual difference in overall infestation rate for the seven plant species sampled (Figure 2). Although the number of samples collected was roughly the same between years, the percentage of *C. amomum* samples infested and the infestation rate was much lower in 2014 than in 2013; the factors contributing to this observed variation are unclear, but consistent with another wild host study [11].

### 3.2. Trapping 

**Figure 3:**
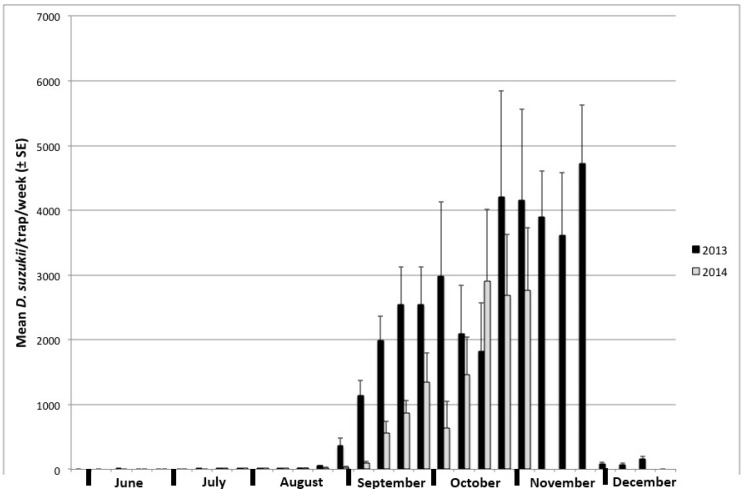
Trapping results of both male and female *D. suzukii*, shown in the average number of flies collected per week per trap in 2013 (black bars) and 2014 (gray bars).

The first adult capture of *D. suzukii* in 2013 was on 5 July, and on 16 July in 2014. Trapping data show a marked increase in captures about three weeks after peak infestation occurred in wild hosts (Figure 3), a lag consistent with *D. suzukii* development rates [29]. This trend was present both years and mirrored the availability of non-crop hosts. For example, the peak infestation period in 2013 lasted about three weeks, but only one week in 2014, and the initial pulse in trapping results reflect this observation. Traps from October and November captured the most flies, in line with seasonal monitoring results reported elsewhere [30,31]. Trap catch was significantly different between years (*p* < 0.001, *F*_1, 367_ = 22.92). The baits used in these traps consistently captured more males than females, with a male:female ratio of 1.24:1.

## 4. Discussion

**Figure 4:**
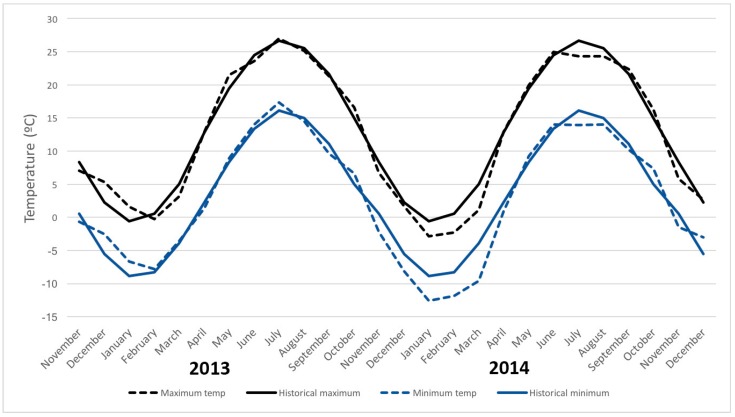
Monthly high (black lines) and low temperatures (blue lines) in Geneva, NY. Solid lines reflect historical averages while dotted lines are the actual reported monthly averages for 2013 and 2014. Data as reported in [32].

Both the similarities and differences between the two sampling years indicate potentially important insights into *D. suzukii* biology and behavior. The principal difference between 2013 and 2014 results were the winter climate that preceded them, the 2012–2013 winter being largely in line with yearly averages. It was unseasonably cold from November 2013 to March 2014, with monthly low temperatures several degrees lower than average and a total snowfall of 1.88 m, as compared to an average annual snowfall of 1.37 m for the Geneva, NY region (Figure 4, Table S3). This severe weather likely affected the number of surviving overwintering adult *D. suzukii* [17,20] and delayed the emergence and growth rates of host plants. For example, in 2014, early-season plants such as *R. allegheniensis* and *R. occidentalis* began fruiting three weeks later than in 2013. The 2014 fruiting season was in general shorter than 2013, as plants fruited later but senesced around the same time, meaning there was a shorter time of ripe fruit available in the second year for several hosts. This affected the weekly infestation results although there was no effect on the average yearly infestation rate. Later fruiting plants were only delayed by one week as compared to the previous year. The week-to-week infestation pattern differences imply that climatic factors may affect not only fly survival, but also how the pest interacts with its host plants. 

Despite some differences, the *D. suzukii* infestation pattern in non-crop hosts remained temporally consistent. Although fewer flies were captured in monitoring traps in 2014, the overall contributions to adult populations by specific plant species did not differ between years. While adult monitoring traps alone cannot measure absolute population levels, the marked difference in relative trap catch raises some interesting questions about the role of fly density on *D. suzukii* population dynamics. Since this study only assessed the number of flies emerging from fruits and not the number of eggs laid, we cannot say whether the total egg number was different between years with certainty. At the larval stage, nutrition and density appear to influence development, with larval survival negatively correlated with density [33]. Moreover, host quality may play a role in limiting larval development [34]. It is likely that fruits from certain species can support only a limited number of offspring to adulthood, as was shown for *P. americana* and cultivated blackberries [34], however further research will need to assess the relationship between egg density, larval survival, and host quality. 

Little is currently known about the effects of fly density on adult behavior. The results from this study suggest the contributions from *D. suzukii* population sources such as non-crop—and potentially crop—host species may be density independent. In *D. melanogaster*, female fecundity is negatively correlated with density [35], where less dense populations tended to have higher fitness (i.e., production of reproductive offspring) than more dense populations. Researchers will need to assess the effects of density on oviposition and fitness in *D. suzukii* specifically. The lower relative 2014 adult trap catch could suggest that, even when pest densities are low, growers may still have to adhere to a strict insecticide spray schedule to adequately protect their crop. 

Although in New York we observed a consistent hierarchical infestation pattern among sampled plants, the relative infestation rates among non-crop hosts in this study differ slightly from other studies (e.g., [11,12,23]), suggesting additional spatial, temporal, and ecological factors may influence infestation patterns. For example, under lab conditions, the European group Poyet et al. (2015) found field-collected *S. dulcamara* to produce more adults by weight than *P. americana* [36], opposite the trend we observed. However, our results align more closely with a U.S.-based wild host survey conducted in the middle (Michigan) and western (Oregon) parts of the country [11]. Lee et al. (2015) [11] found similar infestation rates and timings for several species, including *P. americana*, *Rubus* spp., and *Prunus* spp., although the *Lonicera* fruits sampled in those areas were not as heavily infested as we observed in central New York [11]. Several biotic and abiotic factors may contribute to these disparities: geography, plant and microbial community composition; spatial distribution of wild hosts; and host phenology of crop and non-crop hosts vary on local and regional scales.

As this study shows, early fruiting non-crop plants escape *D. suzukii* infestation, even though *R. occidentalis* is a known host and both *L. morrowii* and *R. allegheniensis* samples were later heavily infested. The cold winter preceding the 2014 growing season only delayed *D. suzukii* infestation by about a week and tracked with the later fruiting times of the sampled plant species. *D. suzukii* infestation was not detected until mid to late July in both years, indicating flies are not using farm-adjacent areas to build populations to later disperse into crop hosts. We were unable to identify a candidate non-crop host to function as an early season indicator of local *D. suzukii* presence (i.e., a sentinel host species). Although the plants sampled were abundant in our study area, we cannot exclude the possibility that other alternate springtime hosts may support emerging overwintered flies. However, the lack of infestation in known and locally abundant hosts such as *Rubus* spp. suggest long distance movement from warmer areas may be a more significant factor in establishing yearly *D. suzukii* populations than local overwintering adults. Ongoing research on overwintering and dispersal capabilities could help answer where springtime adults originate.

Our results align with those of Pelton et al. [37], who found that wooded landscapes near crops in the Upper Midwest of the U.S. were associated with early season *D. suzukii* trap capture, but had no effect on the abundance of *D. suzukii* or overall pest pressure. In our study area, the largest effects from non-crop areas materialized in the middle of August, when the highest numbers of *D. suzukii*-susceptible hosts were available. As suggested by the number of trap captures and emerging adults per fruit sample, population density spiked during this period. Many non-crop plants stop fruiting in early September, leaving female *D. suzukii* searching for appropriate oviposition sites. This high density of flies may encourage dispersal into later fruiting crops, such as fall raspberries, figs, and day neutral strawberries—all susceptible to *D. suzukii*—making fruit protection from infestation more difficult.

The importance of these wild-growing plants for growers may also depend on where these plants are located. The cornfield location was not located near any *D. suzukii*-susceptible crop fields, but was similarly infested as compared to the commercial fruit production sites (data not shown). There is still a large gap in our knowledge of the extent of this fly’s movement and dispersal capabilities. Other *Drosophila* spp. are known to move kilometers in a matter of days [38,39,40]; it is possible adult *D. suzukii* infesting crop plants may have emerged from distantly located sources. Many of the wild hosts sampled in this study are common in disturbed areas along the edges of crop fields and roadsides. The specific species sampled in New York have congeners throughout the United States [41] and other countries [12] and may have substantial roles in *D. suzukii* population dynamics in these regions.

How our results can apply to on-farm management practices is less clear. Although non-crop host plants can clearly serve as a *D. suzukii* population source, it is also likely that some plants may function as population sinks. As Poyet et al. (2015) demonstrated, only a small percentage of eggs laid into species such as *P. americana* and *R. cathartica* survive to adulthood [36]. Regardless of their effect on *D. suzukii* density, these crop-adjacent areas provide additional benefits to growers. Wooded areas containing non-crop *D. suzukii* hosts near crop fields serve as important refuge sites for insecticide resistance management (IRM). *D. suzukii* is a crepuscular species, with some flies moving between wooded edge and crop habitat during morning and evening hours [13,15]. Current *D. suzukii* management programs in conventional and organic systems rely primarily on a calendar-based insecticide rotation whenever ripe fruit is available, and there is concern resistance to certain classes of insecticides may develop. Left unmanaged, field edges may help delay the development of resistance, a vital component of IRM [42,43]. Field edges are also important in the ecology and conservation of many beneficial insects, providing vital habitat and resources for the persistence of natural enemies and pollinators [44,45,46,47,48]. These areas may be key refuge points for beneficial insects, as the use of primarily non-selective insecticides to control *D. suzukii* [49,50] have likely affected the natural enemy community as well. These areas will be an important consideration in the release plans of any potential biocontrol parasitoid. Further research into the contribution of individual plants and communities into local *D. suzukii* infestation will help inform a benefit/cost analysis as to the relative risk of these unmanaged areas.

## Figures and Tables

**Table 1:**
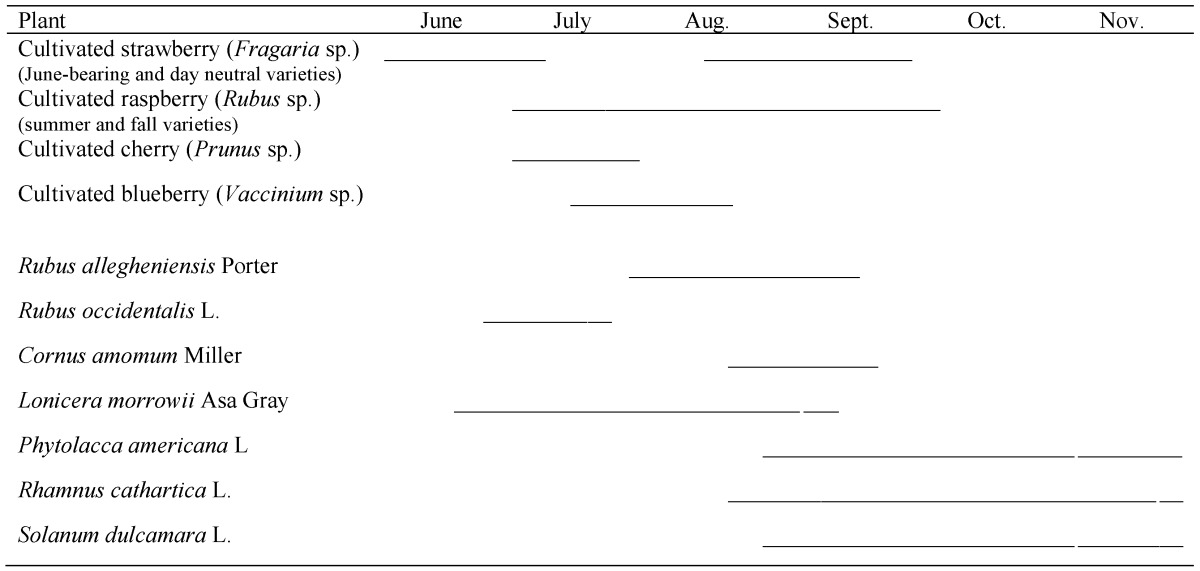
Seasonal phenology of crop and non-crop *D. suzukii* host plants.

**Table 2:** Monthly breakdown of infestation rate for plants sampled plants in 2013 and 2014. Percent of samples infested was calculated by dividing the number of fruit samples from which at least one *D. suzukii* adult emerged. A sample is defined as ~50 fruits each for *R. allegheniensis* and *R. occidentalis*, and 100 fruits for non-*Rubus* hosts.

Month	Host Species	Number of Samples		Percent of Samples Infested		Infestation Rate ± SE (Flies/kg Fruit)	
		2013	2014	2013	2014	2013	2014
June	*L. morrowii*	15	--	0	--	0 ± 0	--
July	*R. allegheniensis*	3	--	66.7	--	28 ± 18	--
	*R. occidentalis*	46	46	6.5	2.2	2.4 ± 1.5	1.1 ± 1.1
	*L. morrowii*	141	50	23.4	6	47 ± 13	1.4 ± 0.8
August	*R. allegheniensis*	73	32	91.7	84.4	1167 ± 161	923 ± 177
	*R. occidentalis*	--	1	--	100	--	84
	*C. amomum*	17	15	64.7	13.3	984 ± 268	163 ± 158
	*L. morrowii*	99	38	85.9	84.2	1313 ± 126	1322 ± 231
	*P. americana*	17	2	76.5	0	1208 ± 256	0 ± 0
	*R. cathartica*	25	6	28	0	23 ± 12	0 ± 0
	*S. dulcamara*	--	20	--	40	--	55 ± 24
September	*R. allegheniensis*	5	1	100	100	1198 ± 259	3913
	*C. amomum*	8	12	87.5	25	1895 ± 466	120 ± 78
	*L. morrowii*	3	2	100	50	2145 ± 270	536 ± 536
	*P. americana*	59	35	89.8	97.1	308 ± 34	479 ± 62
	*R. cathartica*	60	40	63.3	67.5	157 ± 33	135 ± 29
	*S. dulcamara*	11	32	72.7	37.5	64 ± 18	81 ± 32
October	*P. americana*	108	50	74.1	58	151 ± 18	65 ± 13
	*R. cathartica*	82	61	68.3	45.9	105 ± 19	77 ± 17
	*S. dulcamara*	17	41	52.9	12.2	37 ± 11	15 ± 7
November	*P. americana*	70	38	5.7	2.6	2 ± 1.1	0.8 ± 0.8
	*R. cathartica*	61	38	13.1	2.6	8 ± 4	0.7 ± 0.7
	*S. dulcamara*	5	23	0	0	0 ± 0	0 ± 0

## Supplementary Materials

The following are available online at www.mdpi.com/2075-4450/9/1/5/s1, Table S1: Yearly mean infestation rate (*D. suzukii* per gram fruit ± SE) of all plants sampled in 2013, Table S2. Monthly breakdown of infestation in hosts only collected in 2013, Table S3: Climate data showing actual and historical monthly average temperatures and snowfall.
