# Supplementary material for: Non-Crop Host Sampling Yields Insights into Small-Scale Population Dynamics of Drosophila suzukii (Matsumura)
Source: Insects. 2018 Jan 3;9(1):5. doi: 10.3390/insects9010005 (PMC5872270; doi:10.3390/insects9010005)
Supplement: Supplementary file 1 [file insects-09-00005-s001.pdf]

insects

MDPI

*Article*

# Non-Crop Host Sampling Yields Insights into Small-Scale Population Dynamics of *Drosophila suzukii* (Matsumura)

**Supplemental Materials:**

**Table S1.** Yearly mean infestation rate (*D. suzukii* per gram fruit ± SE) of all plants sampled in 2013.

| Host | | No. samples | Infestation rate |
|---|---|---|---|
| **Scientific name** | **Common name** | | |
| *Prunus serotina* | Black cherry | 8 | 0.007 ± 0.007 |
| *Prunus virginiana* | Choke cherry | 23 | 0.003 ± 0.002 |
| *Cornus racemosa* | Gray dogwood | 45 | 0.321 ± 0.087 |
| *Rubus idaeus* | Red raspberry | 11 | 0.004 ± 0.004 |
| *Viburnum opulus* | European cranberrybush | 55 | 0 |

**Table S2.** Monthly break down of hosts only collected in 2013.

| Month | Host species | Number of samples 2013 | Percentage of samples infested 2013 | Infestation rate ± SE (flies/kg fruit) 2013 |
|---|---|---|---|---|
| July | *R, idaeus* | 11 | 9.1 | 3.8 ± 3.8 |
| | *P. virginiana* | 7 | 0 | 0 ± 0 |
| | *C. racemosa* | 4 | 0 | 0 ± 0 |
| August | *C. racemosa* | 22 | 22.7 | 60.4 ± 44.3 |
| | *P. serotina* | 5 | 20 | 11.9 ± 11.9 |
| | *P. virginiana* | 16 | 12.5 | 3.8 ± 2.6 |
| September | *C. racemosa* | 21 | 81 | 553.1 ± 164.5 |
| | *P. serotina* | 3 | 0 | 0 ± 0 |
| | *V. opulus* | 10 | 0 | 0 ± 0 |
| October | *C. racemosa* | 3 | 100 | 505.9 ± 160.8 |
| | *V. opulus* | 27 | 0 | 0 ± 0 |
| November | *V. opulus* | 18 | 0 | 0 ± 0 |

**Table S3.** Climate data showing actual and historical average temperatures and snowfall. Monthly weather averages for Geneva, NY from Nov 2012 to December 2014. All temperatures are in °F, and precipitation is reported in inches.

| Month | Year | Mean high temperature | Historical average high temp | Mean low temperature | Historical average low temp | Total precipitation | Typical monthly precipitation | Snowfall total | Typical monthly snowfall total |
|---|---|---|---|---|---|---|---|---|---|
| November | 2012 | 44.6 | 47 | 30.8 | 33 | 0.81 | 2.87 | 0 | 4 |
| December | 2012 | 41.6 | 36 | 27.5 | 22 | 4.59 | 2.2 | 20.71 | 11 |
| January | 2013 | 34.9 | 31 | 20 | 16 | 0.96 | 1.69 | 5.95 | 14 |
| February | 2013 | 31.5 | 33 | 17.9 | 17 | 1.78 | 1.5 | 15.12 | 11 |
| March | 2013 | 37.7 | 41 | 25.6 | 25 | 0.57 | 2.36 | 3.74 | 12 |
| April | 2013 | 55.2 | 55 | 34.4 | 36 | 3.15 | 2.83 | 0.12 | 2 |
| May | 2013 | 70.6 | 67 | 48.1 | 47 | 3.84 | 3.07 | 0 | 0 |
| June | 2013 | 74.4 | 76 | 57.2 | 56 | 5.8 | 3.66 | 0 | 0 |
| July | 2013 | 80.6 | 80 | 63.2 | 61 | 4.68 | 3.46 | 0 | 0 |

| | | | | | | | | | |
|---|---|---|---|---|---|---|---|---|---|
| August | 2013 | 77.3 | 78 | 58.1 | 59 | 4.05 | 3.03 | 0 | 0 |
| September | 2013 | 70.3 | 71 | 49.3 | 52 | 1.85 | 3.46 | 0 | 0 |
| October | 2013 | 61.9 | 59 | 43.9 | 41 | 3.35 | 3.35 | 0 | 0 |
| November | 2013 | 44.1 | 47 | 28 | 33 | 3.49 | 2.87 | 9.21 | 4 |
| December | 2013 | 35.1 | 36 | 17.4 | 22 | 2.55 | 2.2 | 12.51 | 11 |
| January | 2014 | 26.8 | 31 | 9.4 | 16 | 0.96 | 1.69 | 12.96 | 14 |
| February | 2014 | 27.9 | 33 | 10.6 | 17 | 1.83 | 1.5 | 19.56 | 11 |
| March | 2014 | 34 | 41 | 14.7 | 25 | 0.5 | 2.36 | 18.74 | 12 |
| April | 2014 | 55.2 | 55 | 33.5 | 36 | 2.75 | 2.83 | 1.22 | 2 |
| May | 2014 | 67.9 | 67 | 48.6 | 47 | 4.94 | 3.07 | 0 | 0 |
| June | 2014 | 76.9 | 76 | 57.2 | 56 | 3.86 | 3.66 | 0 | 0 |
| July | 2014 | 75.7 | 80 | 57.1 | 61 | 7.31 | 3.46 | 0 | 0 |
| August | 2014 | 75.7 | 78 | 57.2 | 59 | 3.57 | 3.03 | 0 | 0 |
| September | 2014 | 72.2 | 71 | 50.3 | 52 | 1.33 | 3.46 | 0 | 0 |
| October | 2014 | 61.3 | 59 | 45.2 | 41 | 2.79 | 3.35 | 0 | 0 |
| November | 2014 | 42.5 | 47 | 29.4 | 33 | 1.79 | 2.87 | 7.92 | 4 |
| December | 2014 | 36.8 | 36 | 26.5 | 22 | 1.81 | 2.2 | 14.07 | 11 |

© 2018 by the authors. Submitted for possible open access publication under the terms and conditions of the Creative Commons Attribution (CC BY) license (http://creativecommons.org/licenses/by/4.0/).
